# ^18^F-FDG PET Is Not Inferior to ^68^Ga-PSMA PET for Detecting Biochemical Recurrent Prostate Cancer with a High Gleason Score: A Head-to-Head Comparison Study

**DOI:** 10.3390/diagnostics14010007

**Published:** 2023-12-19

**Authors:** Lian Xu, Ruohua Chen, Xiaofeng Yu, Jianjun Liu, Yuetao Wang

**Affiliations:** 1 Department of Nuclear Medicine, The Third Affiliated Hospital of Soochow University, Changzhou 213003, China; xulian@renji.com; 2 Department of Nuclear Medicine, Ren Ji Hospital, School of Medicine, Shanghai Jiao Tong University, Shanghai 200025, China; 19211@renji.com (R.C.); yuxiaofeng@renji.com (X.Y.); ljjsh@sjtu.edu.cn (J.L.); 3 Institute of Clinical Translation of Nuclear Medicine and Molecular Imaging, Soochow University, Changzhou 213003, China

**Keywords:** prostate cancer, ^68^Ga-PSMA, ^18^F-FDG, biochemical recurrence, PET/CT

## Abstract

Previous studies have indicated that ^18^F-fluorodeoxyglucose (^18^F-FDG) positron emission tomography/computed tomography (PET/CT) in biochemical recurrence (BCR) patients with poorly differentiated prostate adenocarcinoma had higher diagnostic sensitivity than those with well differentiated adenocarcinoma, but whether the performance of FDG PET can achieve the effect of prostate-specific membrane antigen (PSMA) PET in BCR patients with a high Gleason score remains poorly understood. This study aimed to compare the efficacies of ^18^F-FDG PET/CT and ^68^Ga-PSMA PET/CT for BCR patients and evaluate whether ^18^F-FDG PET was not inferior to ^68^Ga-PSMA PET for detecting BCR with a high Gleason score. This was a retrospective, head-to-head comparative study completed at Ren Ji Hospital between May 2018 and June 2021. Patients underwent both ^18^F-FDG and ^68^Ga-PSMA PET/CT. The detection rate of BCR at the patient level and at the anatomical region level was evaluated. In total, 145 patients were enrolled in this study. ^18^F-FDG PET/CT (24.1%, 35/145) had lower detection rates than ^68^Ga-PSMA PET/CT (59.3%, 86/145; *p* < 0.001) at the patient level and at any anatomical region (*p* < 0.05). The PSA level (*p* < 0.001, OR = 11.026, 95% CI: 3.214–37.824) and the Gleason score (*p* < 0.001, OR = 20.227, 95% CI: 5.741–71.267) were independent predictive factors of the detection rate on ^18^F-FDG PET/CT, while the PSA level (*p* < 0.001, OR = 4.862, 95% CI: 2.338–10.110) was the only predictor of the detection rate on ^68^Ga-PSMA PET/CT. ^18^F-FDG PET/CT had a similar detection rate as ^68^Ga-PSMA PET/CT in patients with a Gleason score of 9 at the patient level (64.3% vs. 71.4%, *p* = 0.567) and any anatomical region (all *p* > 0.05), but ^18^F-FDG PET/CT had a lower detection rate than ^68^Ga-PSMA PET/CT in patients with a Gleason score of 6–8. ^18^F-FDG PET is not inferior to ^68^Ga-PSMA PET for detecting BCR with a Gleason score of 9; therefore, ^18^F-FDG PET/CT could be considered in BCR patients with a Gleason score of 9. However, ^68^Ga-PSMA is a better tracer than ^18^F-FDG in PET/CT for treatment decision making in BCR patients with a Gleason score of 6–8.

## 1. Introduction

Prostate cancer (PCa) is one of the most common malignant tumours in men [1]. Radical prostatectomy is the main treatment, and postoperative biochemical recurrence (BCR) is a major problem for these patients [2]. Determining the location and extent of the lesion is important for guiding subsequent treatment. However, conventional imaging modalities including computed tomography (CT), magnetic resonance imaging (MRI) and whole-body bone scan have poor sensitivity for diagnosing metastatic lesions [3,4,5]. Since its application in 2012, ^68^Ga-prostate-specific membrane antigen (^68^Ga-PSMA) positron emission tomography/computed tomography (PET/CT) has significantly improved the imaging sensitivity in PCa [6,7]. Many studies have shown that the detection efficiency of ^68^Ga-PSMA PET is higher than conventional imaging methods and PET acquisition with several earlier tracers, such as ^18^F-fluorodeoxyglucose (^18^F-FDG), choline and fluciclovine [7,8,9].

Although ^18^F-FDG is useful to detect different types of malignant tumours, it is less frequently used because of its low sensitivity (31–61.6%) for detecting metastatic lesions in PCa [10,11]. Though it is generally believed that ^68^Ga-PSMA is superior to ^18^F-FDG in diagnosing BCR patients based on different cohorts [12,13], few head-to-head comparisons between PSMA and FDG PET/CT in the same cohort have been conducted [14]. In addition, previous studies indicated that ^18^F-FDG PET/CT in BCR patients with poorly differentiated adenocarcinoma had higher diagnostic sensitivity than those with well-differentiated adenocarcinoma [11,15]. Our previous study has confirmed the added diagnostic value of ^18^F-FDG PET/CT in BCR patients with negative ^68^Ga-PSMA PET/CT [16], whether the performance of ^18^F-FDG PET can achieve the effect of ^68^Ga-PSMA PET in BCR patients with a high Gleason score remains largely unknown.

Hence, we directly compared the diagnostic efficiency between ^68^Ga-PSMA and ^18^F-FDG PET in the same cohort with BCR patients. The purpose of our study was to compare the detection rates of ^18^F-FDG and ^68^Ga-PSMA PET/CT at the patient level and at the anatomical region level.

## 2. Materials and Methods

### 2.1. Patients

In total, 170 patients were identified who received both ^18^F-FDG and ^68^Ga-PSMA PET/CT from May 2018 to June 2021 in our institution. Of these, 25 were excluded and 145 patients with BCR were finally included in our study. The inclusion criteria were as follows: patients who (a) had histologically confirmed prostate adenocarcinoma; (b) had BCR after radical prostatectomy with a PSA value > 0.2 ng/mL; (c) underwent ^18^F-FDG and ^68^Ga-PSMA PET/CT at an interval of <14 days; and (d) had available data of the PSA, Gleason score, and previous treatment. Ren Ji Hospital Ethics Committee approved this retrospective study, and the need for informed consent was waived.

### 2.2. ^18^F-FDG and ^68^Ga-PSMA PET/CT

Patients were fasted for 6 h before receiving the ^18^F-FDG injection. The dosage of the ^18^F-FDG injection was 3.7 MBq/kg. Patients were expected to keep quiet for 1 h before ^18^F-FDG PET/CT. The dosage of ^68^Ga-PSMA was 1.85 MBq/kg and the ligand of PSMA was ^68^Ga-PSMA-11. ^68^Ga-PSMA-11 synthesis was performed as previously described [17]. PET/CT scan was conducted 50–60 min after injecting ^68^Ga-PSMA. CT images (3 mm section thickness, automatic milliamp current: 120 kV) were scanned from the upper thigh to skull. The scan time of every bed position was 3 min for PET.

### 2.3. Image Evaluation

Two nuclear medicine physicians with 8 years and 12 years of PET/CT interpretation experience evaluated the images independently. In case of disagreements, a discussion was carried out until they reached consensus. According to the interpretation guidelines [18,19,20,21,22], the experts evaluated the presence of positive lesions in local recurrence (T), pelvic lymph nodes (N), extrapelvic nodes (M1a), bone (M1b), or other organs (M1c). After excluding physiological uptake and other important pitfalls, ^68^Ga-PSMA or ^18^F-FDG positivity was defined as focal avidity greater than the background of the mediastinal blood pool. Patients were considered to have positive PET/CT results if local recurrence, lymph node metastasis, or distant metastasis had positive lesions. PSA measurements, imaging examination (including CT, MRI, whole-body bone scan, or PET/CT re-examination), and biopsies were used for follow-up. We used composite validation including histopathology, PSA decreases after PET-directed radiotherapy, and follow-up imaging to verify these positive results.

### 2.4. Statistical Analysis

The k statistic was used to measure agreement between the two readers for positive detection of FDG or PSMA PET/CT. Comparison of detection rates on FDG and PSMA PET/CT in the low or high Gleason score group was assessed by using the chi-square test. The Mann–Whitney U test was used to compare the lesion SUV_max_, lesion-to-aorta ratios, and lesion-to-muscle ratios at the concordant positive region between FDG and PSMA PET/CT. Multivariate regression analyses were used to predict the detection rate of FDG or PSMA PET/CT and patients with or without a gained detection rate upon comparing PSMA with FDG PET/CT, and PSA doubling time was the confounder adjusted for in the multivariate logistic regression. All data were analysed by SPSS 13.0.

## 3. Results

### 3.1. Patients’ Characteristics

**Figure 1:**
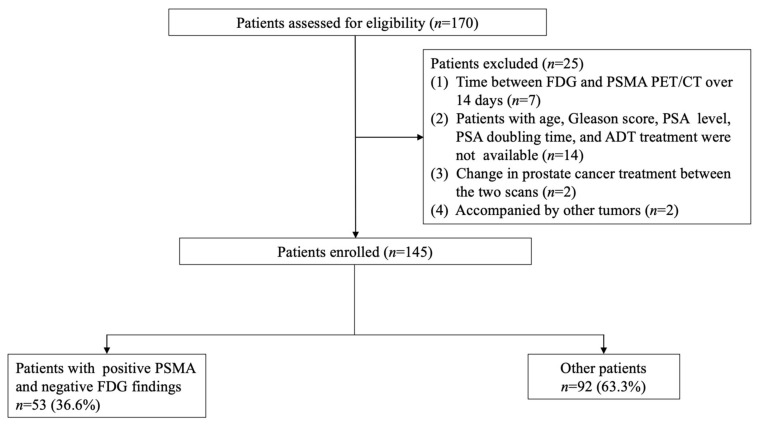
Patient recruitment flowchart.

In total, 170 patients were initially included from June 2018 to June 2021. Of these, 25 were excluded and 145 patients were included for the final analysis (Figure 1). Table 1 shows the patients’ characteristics. The median age was 69 years (IQR: 64–73 years). The number of patients with a Gleason score of 6, 7, 8, and 9 were 7, 72, 38, and 28, respectively. The average PSA was 3.6 ± 0.9 ng/mL. The median interval between ^18^F-FDG and ^68^Ga-PSMA PET/CT was 6.0 days (IQR 1.0–8.0). There were 110 patients (75.9%) who underwent ^18^F-FDG PET/CT before ^68^Ga-PSMA PET/CT, while 35 patients (24.1%) underwent ^18^F-FDG PET/CT after ^68^Ga-PSMA PET/CT. A total of 114 patients (78.6%) received no other treatment after radical prostatectomy, while 31 patients (21.4%) underwent androgen deprivation therapy (ADT) or radiotherapy after surgery. None of the patients experienced any adverse events in the PSMA and FDG PET scan.

### 3.2. Detection Rate of FDG and PSMA PET/CT

**Figure 2:**
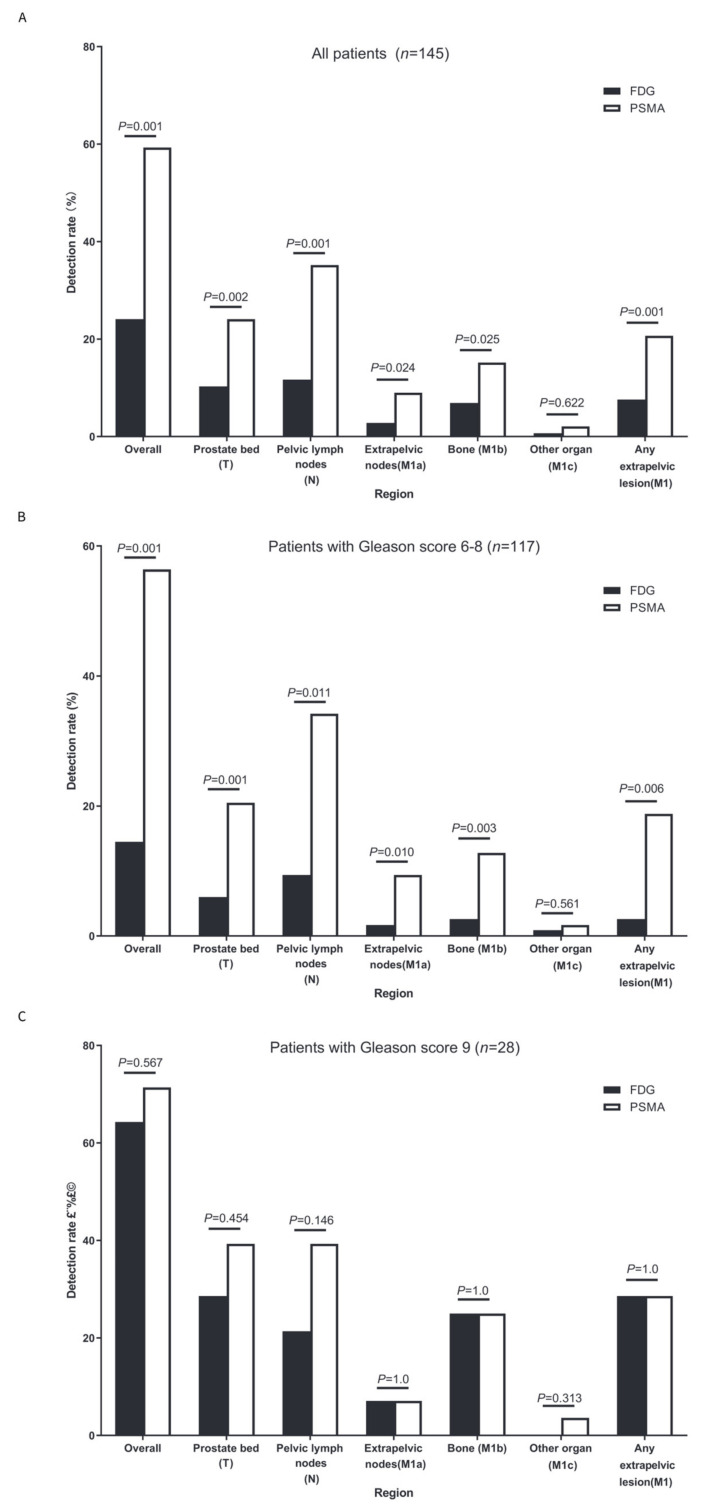
Detection rates per patient and per region. (**A**) Detection rates per patient and per region in the whole cohort (*n* = 145); (**B**) detection rates per patient and per region in patients with a Gleason score of 6–8 (*n* = 117); and (**C**) detection rates per patient and per region in patients with a Gleason score of 9 (*n* = 28).

The detection rate of the BCR per patient was significantly lower with FDG PET/CT (24.1%, 35/145) than with PSMA PET/CT (59.3%, 86/145; *p* < 0.001; Figure 2A).

FDG PET/CT had lower detection rates than PSMA PET/CT for prostate bed recurrence (T) ([10.3%, 15/145] vs. [24.1%, 35/145], *p* = 0.002); pelvic lymph node region (N) ([11.7%, 17/145] vs. [35.2%, 51/145], *p* < 0.001); extrapelvic lymph node region (M1a) ([2.8%, 4/145] vs. [9.0%, 13/145], *p* = 0.024); bone (M1b) ([6.9%, 10/145] vs. [15.2%, 22/145], *p* = 0.025); and any extrapelvic lesions (M1) ([7.6%, 11/145] vs. [20.7%, 30/145], *p* = 0.001) (Figure 2A). No significant differences were found for other organs (M1c) ([0.7%, 1/145] vs. [2.1%, 3/145], *p* = 0.622) (M1a), which was likely due to the sample size not being large enough.

We then performed an exploratory analysis to select predictive factors for positive detection on FDG PET or PSMA PET (Table 2). The optimal PSA threshold (PSA, 0.76 ng/mL) was determined by ROC curve analysis. In the univariable analysis, the PSA level (*p* < 0.001, OR = 7.469, 95% CI: 2.697–20.687) and the Gleason score (*p* < 0.001, OR = 10.588, 95% CI: 4.184–26.793) was associated with positive detection on FDG PET/CT. Multivariable regression analysis revealed that the PSA level (*p* < 0.001, OR = 11.026, 95% CI: 3.214–37.824) and the Gleason score (*p* < 0.001, OR = 20.227, 95% CI: 5.741–71.267) remained as independent predictive factors of positive detection on FDG PET/CT. In addition, the PSA level was the only predictor of positive detection on PSMA PET/CT in univariable analysis (*p* < 0.001, OR = 4.858, 95% CI: 2.379–9.923) and multivariable regression analysis (*p* < 0.001, OR = 4.862, 95% CI: 2.338–10.110).

### 3.3. Differences in Clinical Features between FDG PET-Negative Patients with Positive PSMA PET Results and Other Patients

Among the 145 patients, 34 (23.4%) had positive detection both in PSMA and FDG PET/CT and 53 (36.6%) had only positive detection in PSMA PET/CT. To further investigate which patients could benefit from PSMA PET/CT, all patients were classified into two groups according to whether they benefited from PSMA PET/CT. In total, 53 patients benefited from PSMA PET/CT and 92 patients did not. The results of univariable and multivariable regression analysis for predicting patients who may benefit from PSMA PET/CT are shown in Table 3. The Gleason score was the only significant predictor of likelihood of benefitting from PSMA PET/CT in the univariable (*p* = 0.002, OR = 0.100, 95% CI: 0.023–0.439) and multivariable regression analysis (*p* = 0.002, OR = 0.088, 95% CI: 0.019–0.401). Namely, patients likely to benefit from PSMA PET/CT had lower Gleason scores than those without (7 (7–8) vs. 8 (7–9); *p* < 0.001).

**Figure 3:**
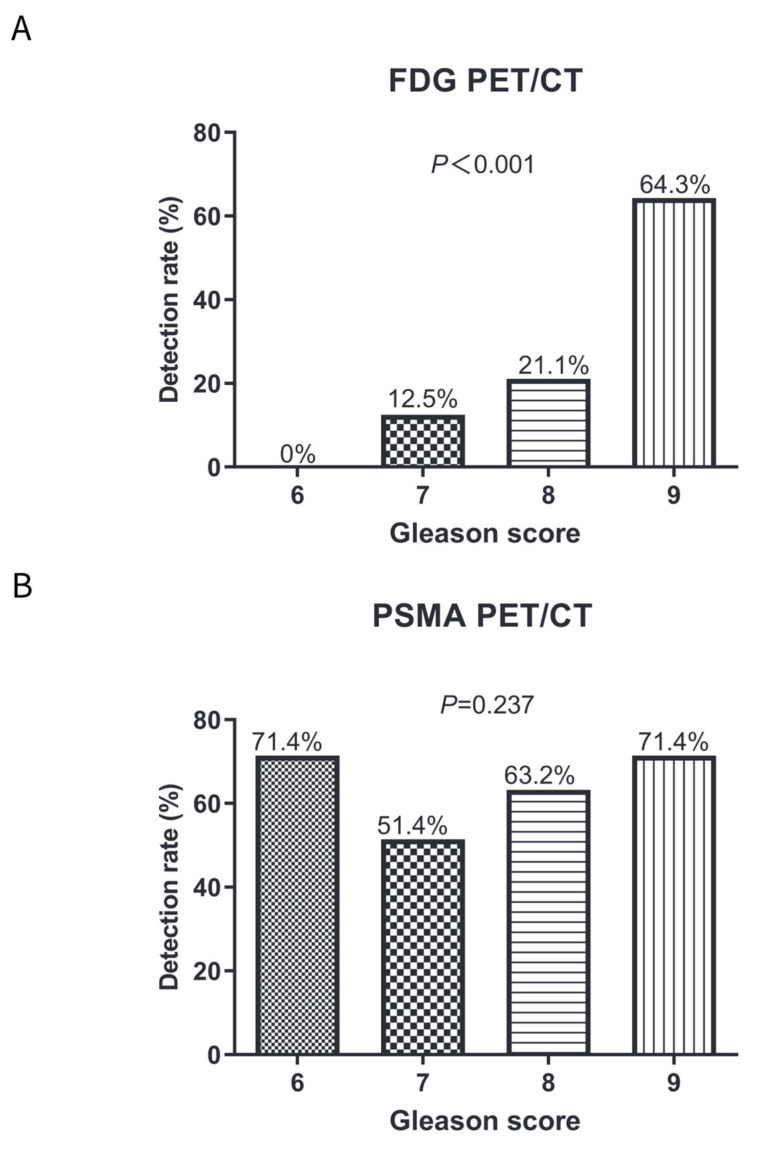

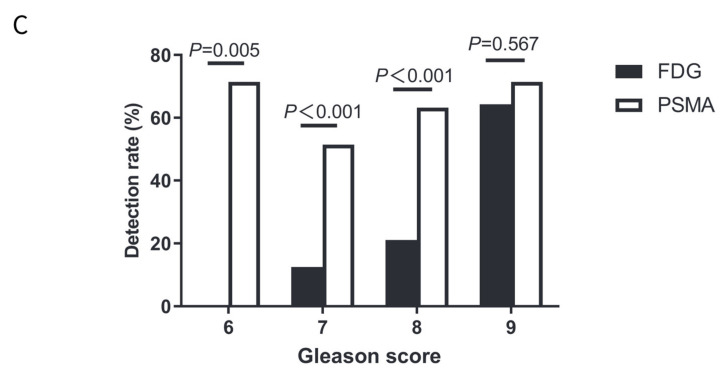
Comparisons of detection rates on FDG and PSMA PET/CT in patients with a Gleason score of 6–8 or a Gleason score of 9. (**A**) Detection rates on FDG PET/CT in patients with Gleason score 6–9; (**B**) Detection rates on PSMA PET/CT in patients with Gleason score 6–9; and (**C**) Comparisons of detection rates on FDG and PSMA PET/CT in patients with different Gleason scores.

For FDG PET/CT, the detection rate in patients with a Gleason score of 6, 7, 8, and 9 was 0% (0/7), 12.5% (9/72), 21.1% (8/38), and 64.3% (18/28), respectively (Figure 3A). There was a positive relationship between Gleason score and the detection rate of FDG PET/CT in BCR patients (Pearson correlation coefficient, 32.397; *p* < 0.001). For PSMA PET/CT, the detection rate of a Gleason score of 6, 7, 8, and 9 was 71.4% (5/7), 51.4% (37/72), 63.2% (24/38), and 71.4% (20/28), respectively (Figure 3B). No significant difference was found in the detection rates of PSMA PET/CT between different Gleason scores (Spearman correlation coefficient, 4.235; *p* = 0.237).

PSMA PET/CT had a higher detection rate than FDG PET/CT in a Gleason score of 6 (71.4% vs. 0%; *p* = 0.005), a Gleason score of 7 (51.4% vs. 12.5%; *p* < 0.001), and a Gleason score of 8 (63.2% vs. 21.1%; *p* < 0.001) (Figure 3C). However, no significant difference was observed between FDG and PSMA PET/CT in patients with a Gleason score of 9 (71.4% vs. 64.3%; *p* = 0.567).

### 3.4. Comparison of Detection Rates of FDG and PSMA PET/CT Based on Lesion Region and Gleason Score

We further compared the detection rates of FDG and PSMA PET/CT based on the lesion region and the Gleason score. For patients with a Gleason score of 6–8 (Figure 2B), FDG PET/CT had lower detection rates than PSMA PET/CT for prostate bed recurrence (T) ([6.0%, 7/117] vs. [20.5%, 24/117], *p* = 0.001); pelvic lymph node region (N) ([9.4%, 11/117] vs. [34.2%, 40/117], *p* = 0.011); extrapelvic lymph node region (M1a) ([1.7%, 2/117] vs. [9.4%,11/117], *p* = 0.010); bone (M1b) ([2.6%, 3/117] vs. [12.8%, 15/117], *p* = 0.003); and any extrapelvic lesions (M1) ([2.6%, 3/117] vs. [18.8%, 22/117], *p* = 0.006). No significant differences were found for other organs (M1c) ([0.9%, 1/117] vs. [1.7%, 2/117], *p* = 0.561), possibly because the sample size was not large enough.

For patients with a Gleason score of 9 (Figure 2C), no significant detection rates were observed between PSMA and FDG PET/CT for the pelvic lesion (prostate bed and pelvic lymph node); individual extrapelvic lesions (M1a, M1b, and M1c); and any extrapelvic lesions (M1) (all *p* > 0.05).

### 3.5. Semiquantitative Analysis of Concordantly Positive Lesions in Patients with a Gleason Score of 9

Among the 28 patients with a Gleason score of 9, 18 had positive detection both in PSMA and FDG PET/CT. Of these 18 patients, eight had concordant positive lesions in the prostate bed, six had concordant positive lesions in the pelvic lymph node region, three had concordant positive lesions in the extrapelvic lymph node region, seven had concordant positive lesions in the bone, none had concordant positive lesions in other organs, and eight had concordant positive lesions in any extrapelvic lesions. The semiquantitative analysis (SUV_max_) and lesion-to-background ratio (L/B) of lesion uptake were evaluated in the concordantly positive lesions (Table 4).

The overall lesion SUV_max_ was lower for FDG than PSMAs (4.8 ± 2.8 vs. 18.5 ± 7.1, *p* < 0.001); the overall lesion-to-aorta ratios were lower for FDG than PSMAs (2.0 ± 0.8 vs. 6.8 ± 3.0, *p* < 0.001); and the overall lesion-to-muscle ratios were lower for FDG than PSMAs (6.7 ± 2.1 vs. 28.0 ± 12.4, *p* < 0.001). However, there was no difference in the overall lesion-to-liver ratios between FDG and PSMAs (1.9 ± 0.6 vs. 3.6 ± 1.3, *p* = 0.635). Similar results were also observed when the overall lesions were replaced with prostate bed (T), pelvic lymph nodes (N), bone (M1b), or any extrapelvic lesions (M1). However, no significant difference was observed between FDG and PSMAs (including SUV_max_, lesion-to-liver ratios, lesion-to-aorta ratios and lesion-to-muscle ratios) for extrapelvic lymph nodes (M1a) (all *p* > 0.05). No concordant positive region was found for other organs (M1c).

### 3.6. Inter-Reader Agreement and Validation of Positive Lesions

FDG PET/CT had lower inter-reader agreement than PSMA PET/CT (κ values: 0.76 vs. 0.84) at the patient level and per region (both *p* < 0.05).

Among the 87 patients with positive PET findings, positive results were confirmed in 23 (26.4%) of 87 patients. Lesion validation included histopathology (*n* = 2), and the PSA decreased after PET-directed radiotherapy (*n* = 21). Details of the 23 patients with lesion validation are shown in Table 5. For the other 64 patients with positive PET findings, 41 patients received systemic treatment, but no follow-up imaging or histopathology was performed; and 23 people did not receive any treatment or examination.

No false-positive results were observed on the two tracers in the 23 patients whose lesions were confirmed. The positive predictive value was 100% for both FDG and PSMA PET/CT. The sensitivity per patient was 43.5% (10/23) for FDG and 100% (23/23) for PSMA PET/CT (*p* < 0.001) (Table 6).

Among the 23 patients whose lesions were verified, 17 had a Gleason score of 6–8 and six had a score of 9 (Table 6). For the 17 patients with a Gleason score of 6–8, the per-patient sensitivity was 35.3% (6/17) for FDG and 100% (17/17) for PSMA PET/CT (*p* < 0.001). For the six patients with a Gleason score of 9, the per-patient sensitivity was 66.7% (4/6) for FDG and 100% (6/6) for PSMA PET/CT (*p* = 0.455).

## 4. Discussion

Superior accuracy has led to wide use of PSMA PET/CT in PCa. However, several studies demonstrated the diagnostic value of FDG PET in PCa patients, performing both FDG and PSMA PET. Wang et al. showed that FDG after PSMA PET improved the detection of metastases from 65% to 73% in high-risk early castration-resistant PCa with negative conventional imaging [23]. Our previous study found that the detection rate per patient for ^18^F-FDG PET/CT was 16.7% in BCR patients with ^68^Ga-PSMA-negative findings, and the PSA and Gleason score correlated with ^18^F-FDG-positive findings. Although previous studies indicated that ^18^F-FDG PET/CT had relatively high diagnostic value in BCR patients with poorly differentiated adenocarcinoma, the performance of FDG PET in BCR patients remains largely unknown. Our study aimed to compare retrospectively paired FDG and PSMA PET/CT scans for the detection rate in BCR patients. In this current study, we retrospectively compared the detection rate at the patient level and anatomical region and evaluated predictive factors for the detection rate on FDG PET/CT or PSMA PET/CT in BCR patients.

Detection rates of FDG and PSMA PET/CT in BCR patients have been reported: they averaged around 8.1–75% for FDG [11] and 56.0–78.5% for PSMA PET/CT [12,24]. Meanwhile, all these studies evaluated the detection rates of the two tests in a different cohort. In this study, we found that the detection rate of PSMA PET/CT was higher than FDG PET/CT (59.3% vs. 24.1%) at the patient level in the same cohort. Detection rates of both tracers in the current study fall within the scope of previously reported studies.

Although previous studies showed that FDG PET/CT had a higher detection rate in BCR patients with poorly differentiated adenocarcinoma [11], the independent predicting factors for its detection rate had never been evaluated. Our study found that in BCR patients, the PSA value and Gleason score were independent predicting factors of the detection rate. For FDG PET/CT, patients with a high PSA value and Gleason score were more likely to have positive detection of BCR, which was similar to previous conclusions in BCR patients [11]. Meanwhile, the PSA value was the only predictor of positive detection on PSMA PET/CT in BCR patients, which was also consistent with previous studies [12].

We further found that the Gleason score was a significant predictor of patients who were likely to benefit from PSMA PET/CT compared to FDG PET/CT, whereas the PSA level was not. FDG PET/CT had a similar detection rate as PSMA PET/CT in patients with a Gleason score of 9, but had lower detection rate than PSMA PET/CT in those with a Gleason score of 6–8. Several factors might account for the non-inferior detection rate of FDG PET/CT compared to PSMA PET/CT in patients with a Gleason score of 9. First, the glucose transporter GLUT1, which transports FDG from the extracellular to the intracellular space, is highly expressed in PCa with a high Gleason score [25]. By contrast, the Gleason score was not associated with the detection rate of PSMA PET/CT in BCR patients [26,27]. Therefore, the increased detection rate of ^18^F-FDG in BCR patients with a high Gleason score could be attributed to the high expression of GLUT1. In addition, the dedifferentiation or neuroendocrine transformation could lead to low PSMA uptake in high-risk PCa patients. Furthermore, neuroendocrine PCa presents a more aggressive course and therefore an expected increased anaerobic glycolysis with consequent FDG uptake [28]. And the correlation between glucose uptake levels of associated genes with neuroendocrine gene signature and low PSMA expression is well-supported [29]. Hence, because FDG PET/CT is not inferior to PSMA PET/CT for detecting BCR with a Gleason score of 9, FDG PET/CT could be considered in BCR patients with a Gleason score of 9, although PSMA is comparatively the better tracer when PET/CT is considered for treatment decision making in BCR patients with a Gleason score of 6–8.

Although no significant detection rates were observed between FDG and PSMA PET/CT at any anatomical region in patients with a Gleason score of 9, the lesion SUV_max_, lesion-to-aorta ratios, and lesion-to-muscle ratios of overall lesions, prostate bed, pelvic lymph nodes, or bone were all lower for FDG than PSMA for the concordantly PET-positive lesions. This result is significant because BCR lesions were most frequently found in the prostate bed, pelvic lymph nodes, and bone involvement; and early detection of prostate bed, pelvic lymph nodes, or bone metastasis is crucial for subsequent treatment such as salvage node dissection or salvage radiotherapy for local lymph node metastases and salvage radiotherapy for prostate bed and solitary bone metastases. The higher overall lesion SUV_max_, lesion-to-aorta ratios, and lesion-to-muscle ratios could also explain why PSMA PET had higher inter-reader agreement than FDG PET both at the patient level and anatomical region level.

It should be noted that there was no significant difference in the lesion-to-liver ratios for overall lesions, prostate bed, pelvic lymph nodes, or bone between FDG and PSMA PET. Several reasons could account for the non-superiority of PSMA PET/CT when liver was used as the reference standard. First, the absolute SUV_max_ of normal liver was higher in PSMA than FDG PET/CT [30]. Second, the absolute SUV_max_ of aorta and muscle were similar between PSMA and FDG PET/CT in our study (*p* = 0.581). Thus, normal liver should not be recommended as the reference standard when semi-quantitative analysis is used for evaluating metastatic lesions.

In patients with early-stage BCR, PET-positive lesions are rarely confirmed by histopathology, because it is often difficult to obtain pathological tissues (such as bone lesions or deep pelvic lymph nodes) [16]. In our study, lesions were validated in 23 of 87 patients. Notably, two of 23 patients (8.7%) received PET-directed surgery; and 21 of 23 patients (91.3%) received PET-directed radiotherapy, with the PSA decreasing after radiotherapy. Among the 23 patients whose lesions were verified, FDG PET/CT had lower sensitivity than PSMA PET/CT in patients with a Gleason score of 6–8 and the sensitivity of the two tests did not differ significantly in patients with a Gleason score of 9. Therefore, the difference in sensitivity between the two scans in these 23 patients whose lesions were verified, was consistent with the results of the whole cohort.

Our study had several limitations, including its relatively small sample size and retrospective design. Because the PET results could not be confirmed by pathology in 84.1% of patients, sensitivity and specificity could not be evaluated in the whole cohort. In addition, our study did not assess the effect of PET/CT on subsequent treatment. Because these two scans were conducted in the same patient within 2 weeks, we could not evaluate the independent effect of the two scans. Therefore, multicentre prospective studies with a greater sample size are needed to further validate our results.

## 5. Conclusions

This retrospective head-to-head comparison between FDG and PSMA PET/CT in 145 BCR patients showed that FDG PET had a lower detection rate than PSMA PET in patients with a Gleason score of 6–8; whereas, FDG PET had a similar detection rate as PSMA PET in patients with a Gleason score of 9 at both the patient level and any anatomical region level. Thus, FDG PET is not inferior to PSMA PET for detecting BCR with a Gleason score of 9 and FDG PET/CT can be considered in BCR patients with a Gleason score of 9, though PSMA is comparatively the better tracer when PET/CT is considered for treatment decision making in BCR patients with Gleason scores of 6–8.

## Figures and Tables

**Table 1:** Characteristics of patients and tumours (*n* = 145).

Characteristics	
**Age (years)**	
Mean ± SD	68.5 ± 6.6
Median (IQR)	69.0 (64.0–73.0)
**PSA doubling time (months)**	
Mean ± SD	8.4 ± 6.1
Median (IQR)	5.8 (3.0–9.1)
**PSA level (ng/mL)**	
Mean ± SD	3.6 ± 0.9
Median (IQR)	0.87 (0.50–2.31)
**Prior therapy**	
None	114
Radiotherapy or ADT	31
**Two scans interval (days)**	
Mean ± SD	5.5 ± 0.4
Median (IQR)	6.0 (1.0–8.0)
**Gleason score**	
6	7
7	72
8	38
9	28

**Table 2:** Univariate and multivariate regression analysis for positive detection on FDG or PSMA PET/CT (*n* = 145).

Variable and Intercept	FDG PET/CT				PSMA PET/CT			
	Univariate Logistic Regression		Multivariate Logistic Regression		Univariate Logistic Regression		Multivariate Logistic Regression	
	OR (95% CI)	*p*	OR (95% CI)	*p*	OR (95% CI)	*p*	OR (95% CI)	*p*
**Age (years)**	0.592 (0.204–1.715)	0.334	0.333 (0.084–1.318)	0.117	1.540 (0.572–4.145)	0.393	1.473 (0.493–4.405)	0.488
**PSA doubling time (months)** (≥6 vs. <6)	0.765 (0.542–1.080)	0.262	0.742 (0.462–1.192)	0.421	0.732 (0.348–1.540)	0.381	0.836 (0.542–1.289)	0.524
**PSA level** (high vs. low)	7.469 (2.697–20.687)	<0.001	11.026 (3.214–37.824)	<0.001	4.858 (2.379–9.923)	<0.001	4.862 (2.338–10.110)	<0.001
**Radiotherapy** (present vs. absent)	2.162 (0.346–13.492)	0.409	5.269 (0.341–81.521)	0.234	2.829 (0.308–25.971)	0.358	3.760 (0.362–39.036)	0.267
**ADT therapy** (present vs. absent)	0.931 (0.341–2.540)	0.889	0.460 (0.110–1.918)	0.287	0.630 (0.269–1.478)	0.288	0.611 (0.230–1.620)	0.322
**Two scans interval (days)**	1.083 (0.502–2.339)	0.839	0.749 (0.274–2.046)	0.573	0.933 (0.477–1.828)	0.841	0.762 (0.356–1.630)	0.484
**Gleason score** (high vs. low)	10.588 (4.184–26.793)	<0.001	20.227 (5.741–71.267)	<0.001	1.358 (0.903–2.012)	0.144	2.013 (0.732–5.538)	0.175

**Table 3:** Univariate and multivariate regression to predict patients with or without a gained detection rate upon comparing PSMA with FDG PET/CT (*n* = 145).

Variable and Intercept	Univariate Logistic Regression		Multivariate Logistic Regression	
	OR (95% CI)	*p*	OR (95% CI)	*p*
**Age (years)**	2.199 (0.684–7.065)	0.186	2.340 (0.700–7.823)	0.168
**PSA doubling time (months)**	0.834 (0.634–1.097)	0.325	0.865 (0.532–1.406)	0.436
(≥6 vs. <6)				
**PSA level**	1.459 (0.735–2.896)	0.280	1.703 (0.815–3.558)	0.157
(high vs. low)				
**Radiotherapy**	1.163 (0.188–7.195)	0.871	1.307 (0.179–9.524)	0.792
(present vs. absent)				
**ADT therapy**	0.585 (0.228–1.500)	0.264	0.883 (0.312–2.496)	0.815
(present vs. absent)				
**Two scans interval (days)**	1.008 (0.508–2.003)	0.981	1.135 (0.537–2.400)	0.741
**Gleason score**	0.100 (0.023–0.439)	0.002	0.088 (0.019–0.401)	0.002
(high vs. low)				

**Table 4:** Semiquantitative analysis of concordant positive regions (*n* = 18).

Concordant Positive Region	*n*	Leison SUV_max_			L/B Liver			L/B Aorta			L/B Muscle		
		FDG	PSMA	*p*	FDG	PSMA	*p*	FDG	PSMA	*p*	FDG	PSMA	*p*
Overall	18	4.8 ± 2.8	18.5 ± 7.1	<0.001	1.9 ± 0.6	3.6 ± 1.3	0.635	2.0 ± 0.8	6.8 ± 3.0	<0.001	6.7 ± 2.1	28.0 ± 12.4	<0.001
Prostate bed (T)	8	4.6 ± 1.3	19.3 ± 6.3	0.007	1.5 ± 0.4	3.6 ± 1.2	0.130	2.1 ± 0.6	8.8 ± 2.9	0.038	6.5 ± 1.9	27.6 ± 9.0	0.038
Pelvic LN (N)	6	2.6 ± 0.3	17.1 ± 8.1	0.002	0.9 ± 0.1	3.2 ± 1.5	0.180	1.2 ± 0.1	7.8 ± 3.7	0.022	3.7 ± 0.4	24.4 ± 11.5	0.040
Extrapelvic LN (M1a)	3	6.9 ± 4.4	6.7 ± 4.8	0.995	2.4 ± 1.6	1.4 ± 1.0	0.649	3.0 ± 2.0	3.1 ± 2.1	0.976	9.1 ± 6.1	9.0 ± 5.9	0.985
Bone (M1b)	7	5.2 ± 1.9	21.6 ± 11.6	0.009	1.8 ± 0.8	3.8 ± 2.3	0.059	1.9 ± 1.0	5.0 ± 3.4	0.036	7.4 ± 2.7	30.9 ± 16.6	0.003
Other organs (M1c)	0	N/A	N/A	N/A	N/A	N/A	N/A	N/A	N/A	N/A	N/A	N/A	N/A
Any extrapelvic lesions (M1)	8	5.5 ± 2.8	18.4 ± 12.2	0.007	1.9 ± 0.9	3.5 ± 2.3	0.079	2.5 ± 1.3	8.3 ± 5.6	0.014	7.9 ± 4.1	26.2 ± 17.4	0.007

LN: lymph nodes; L/B: lesion-to-background ratio; and values are mean ± SD; N/A: not applicable.

**Table 5:** Details of the 23 patients with lesion validation (*n* = 23).

Patient	PSA	Gleason Score	FDG Scan	FDG Final Diagnosis	PSMA Scan	PSMA Final Diagnosis	Region Validated	Lesion Validated	Validation Method
1	1.29	7	T0N0M0	FN	T0N1M0	TP	N1	Iliac lymph nodes	Histopathology
2	0.86	8	T0N0M0	FN	T0N1M0	TP	N1	Iliac lymph nodes	Histopathology
3	0.78	8	T0N0M0	FN	TrN0M0	TP	Tr	Prostate Fossa	PSA decreases after focal radiotherapy
4	1.86	7	T0N0M0	FN	T0N0M1b	TP	M1b	Bone	PSA decreases after focal radiotherapy
5	0.49	8	T0N0M0	FN	T0N0M1b	TP	M1b	Bone	PSA decreases after focal radiotherapy
6	7.90	9	T0N0M1b	TP	T0N0M1b	TP	M1b	Bone	PSA decreases after focal radiotherapy
7	0.83	7	TrN0M0	TP	TrN0M0	TP	Tr	Prostate Fossa	PSA decreases after focal radiotherapy
8	1.30	9	T0N0M1b	TP	TrN1M1b	TP	Tr	Prostate Fossa	PSA decreases after focal radiotherapy
9	0.46	8	T0N0M0	FN	T0N1M0	TP	N1	Iliac lymph nodes	PSA decreases after focal radiotherapy
10	1.59	8	T0N1M0	TP	T0N1M0	TP	N1	Iliac lymph nodes	PSA decreases after focal radiotherapy
11	0.56	7	T0N0M0	FN	TrN1M1b	TP	Tr	Prostate Fossa	PSA decreases after focal radiotherapy
12	0.64	8	T0N0M1b	TP	T0N0M1b	TP	M1b	Bone	PSA decreases after focal radiotherapy
13	3.80	7	T0N0M0	FN	TrN0M0	TP	Tr	Prostate Fossa	PSA decreases after focal radiotherapy
14	0.92	7	TrN0M0	TP	TrN0M0	TP	Tr	Prostate Fossa	PSA decreases after focal radiotherapy
15	12.72	8	T0N0M0	FN	TrN0M0	TP	Tr	Prostate Fossa	PSA decreases after focal radiotherapy
16	0.22	8	T0N0M0	FN	T0N1M0	TP	N1	Iliac lymph nodes	PSA decreases after focal radiotherapy
17	1.55	9	T0N1M0	TP	T0N1M0	TP	N1	Iliac lymph nodes	PSA decreases after focal radiotherapy
18	6.19	9	T0N0M0	FN	T0N1M0	TP	N1	Iliac lymph nodes	PSA decreases after focal radiotherapy
19	0.66	9	T0N0M0	FN	TrN0M0	TP	Tr	Prostate Fossa	PSA decreases after focal radiotherapy
20	1.53	7	TrN0M0	TP	TrN0M0	TP	Tr	Prostate Fossa	PSA decreases after focal radiotherapy
21	0.81	9	TrN0M0	TP	TrN0M0	TP	Tr	Prostate Fossa	PSA decreases after focal radiotherapy
22	3.28	7	T0N0M0	FN	T0N0M1b	TP	M1b	Bone	PSA decreases after focal radiotherapy
23	1.14	7	TrN0M0	TP	TrN0M0	TP	Tr	Prostate Fossa	PSA decreases after focal radiotherapy

Tr: local recurrence; TP: True positive; and FN: False negative.

**Table 6:** Per-patient sensitivity for the patients with lesion validation (*n* = 23).

Patients	FDG			PSMA			
	TP	FN	Sensitivity	TP	FN	Sensitivity	*p*
Total patients (*n* = 23)	10	13	43.5%	23	0	100.0%	<0.001
Low Gleason score (*n* = 17)	6	11	35.3%	17	0	100.0%	<0.001
High Gleason score (*n* = 6)	4	2	66.7%	6	0	100.0%	0.455

TP: True positive; FN: False negative.

## Data Availability

The data that support the findings of this study are available on request from the corresponding author, Y.W., upon reasonable request.

## References

[1] Siegel R.L., Miller K.D., Fuchs H.E., Jemal A. (2022). Cancer statistics, 2022. CA Cancer J. Clin..

[2] Boorjian S.A., Eastham J.A., Graefen M., Guillonneau B., Karnes R.J., Moul J.W., Schaeffer E.M., Stief C., Zorn K.C. (2012). A critical analysis of the long-term impact of radical prostatectomy on cancer control and function outcomes. Eur. Urol..

[3] Briganti A., Abdollah F., Nini A., Suardi N., Gallina A., Capitanio U., Bianchi M., Tutolo M., Passoni N.M., Salonia A. (2012). Performance characteristics of computed tomography in detecting lymph node metastases in contemporary patients with prostate cancer treated with extended pelvic lymph node dissection. Eur. Urol..

[4] Harisinghani M.G., Barentsz J., Hahn P.F., Deserno W.M., Tabatabaei S., van de Kaa C.H., de la Rosette J., Weissleder R. (2003). Noninvasive detection of clinically occult lymph-node metastases in prostate cancer. N. Engl. J. Med..

[5] Hövels A.M., Heesakkers R.A., Adang E.M., Jager G.J., Strum S., Hoogeveen Y.L., Severens J.L., Barentsz J.O. (2008). The diagnostic accuracy of CT and MRI in the staging of pelvic lymph nodes in patients with prostate cancer: A meta-analysis. Clin. Radiol..

[6] Perera M., Papa N., Christidis D., Wetherell D., Hofman M.S., Murphy D.G., Bolton D., Lawrentschuk N. (2016). Sensitivity, Specificity, and Predictors of Positive ^68^Ga-Prostate-specific Membrane Antigen Positron Emission Tomography in Advanced Prostate Cancer: A Systematic Review and Meta-analysis. Eur. Urol..

[7] Perera M., Papa N., Roberts M., Williams M., Udovicich C., Vela I., Christidis D., Bolton D., Hofman M.S., Lawrentschuk N. (2020). Gallium-68 Prostate-specific Membrane Antigen Positron Emission Tomography in Advanced Prostate Cancer-Updated Diagnostic Utility, Sensitivity, Specificity, and Distribution of Prostate-specific Membrane Antigen-avid Lesions: A Systematic Review and Meta-analysis. Eur. Urol..

[8] Afshar-Oromieh A., Zechmann C.M., Malcher A., Eder M., Eisenhut M., Linhart H.G., Holland-Letz T., Hadaschik B.A., Giesel F.L., Debus J. (2014). Comparison of PET imaging with a (68)Ga-labelled PSMA ligand and (18)F-choline-based PET/CT for the diagnosis of recurrent prostate cancer. Eur. J. Nucl. Med. Mol. Imaging.

[9] Rayn K.N., Elnabawi Y.A., Sheth N. (2018). Clinical implications of PET/CT in prostate cancer management. Transl. Androl. Urol..

[10] Öztürk H., Karapolat I. (2016). (18)F-fluorodeoxyglucose PET/CT for detection of disease in patients with prostate-specific antigen relapse following radical treatment of a local-stage prostate cancer. Oncol. Lett..

[11] Jadvar H. (2013). Imaging evaluation of prostate cancer with ^18^F-fluorodeoxyglucose PET/CT: Utility and limitations. Eur. J. Nucl. Med. Mol. Imaging.

[12] Crocerossa F., Marchioni M., Novara G., Carbonara U., Ferro M., Russo G.I., Porpiglia F., Di Nicola M., Damiano R., Autorino R. (2021). Detection Rate of Prostate Specific Membrane Antigen Tracers for Positron Emission Tomography/Computerized Tomography in Prostate Cancer Biochemical Recurrence: A Systematic Review and Network Meta-Analysis. J. Urol..

[13] Fendler W.P., Calais J., Eiber M., Flavell R.R., Mishoe A., Feng F.Y., Nguyen H.G., Reiter R.E., Rettig M.B., Okamoto S. (2019). Assessment of 68Ga-PSMA-11 PET Accuracy in Localizing Recurrent Prostate Cancer: A Prospective Single-Arm Clinical Trial. JAMA Oncol..

[14] McGeorge S., Kwok M., Jiang A., Emmett L., Pattison D.A., Thomas P.A., Yaxley J.W., Roberts M.J. (2021). Dual-Tracer Positron-Emission Tomography Using Prostate-Specific Membrane Antigen and Fluorodeoxyglucose for Staging of Prostate Cancer: A Systematic Review. Adv. Urol..

[15] Richter J.A., Rodríguez M., Rioja J., Peñuelas I., Martí-Climent J., Garrastachu P., Quincoces G., Zudaire J., García-Velloso M.J. (2010). Dual tracer 11C-choline and FDG-PET in the diagnosis of biochemical prostate cancer relapse after radical treatment. Mol. Imaging Biol..

[16] Chen R., Wang Y., Shi Y., Zhu Y., Xu L., Huang G., Liu J. (2021). Diagnostic value of ^18^F-FDG PET/CT in patients with biochemical recurrent prostate cancer and negative 68Ga-PSMA PET/CT. Eur. J. Nucl. Med. Mol. Imaging.

[17] Demirci E., Sahin O.E., Ocak M., Akovali B., Nematyazar J., Kabasakal L. (2016). Normal distribution pattern and physiological variants of ^68^Ga-PSMA-11 PET/CT imaging. Nucl. Med. Commun..

[18] Eiber M., Herrmann K., Calais J., Hadaschik B., Giesel F.L., Hartenbach M., Hope T., Reiter R., Maurer T., Weber W.A. (2018). Prostate Cancer Molecular Imaging Standardized Evaluation (PROMISE): Proposed miTNM Classification for the Interpretation of PSMA-Ligand PET/CT. J. Nucl. Med..

[19] Seifert R., Emmett L., Rowe S.P., Herrmann K., Hadaschik B., Calais J., Giesel F.L., Reiter R., Maurer T., Heck M. (2023). Second Version of the Prostate Cancer Molecular Imaging Standardized Evaluation Framework Including Response Evaluation for Clinical Trials (PROMISE V2). Eur. Urol..

[20] Hofman M.S., Hicks R.J., Maurer T., Eiber M. (2018). Prostate-specific Membrane Antigen PET: Clinical Utility in Prostate Cancer, Normal Patterns, Pearls, and Pitfalls. Radiographics.

[21] Fendler W.P., Eiber M., Beheshti M., Bomanji J., Ceci F., Cho S., Giesel F., Haberkorn U., Hope T.A., Kopka K. (2017). (68)Ga-PSMA PET/CT: Joint EANM and SNMMI procedure guideline for prostate cancer imaging: Version 1.0. Eur. J. Nucl. Med. Mol. Imaging.

[22] Clark M.S., Packard A.T., Johnson D.R., Johnson G.B. (2019). Pitfalls of a Mixed Metabolic Response at PET/CT. Radiographics.

[23] Wang B., Liu C., Wei Y., Meng J., Zhang Y., Gan H., Xu X., Wan F., Pan J., Ma X. (2020). A Prospective Trial of ^68^Ga-PSMA and ^18^F-FDG PET/CT in Nonmetastatic Prostate Cancer Patients with an Early PSA Progression During Castration. Clin. Cancer Res..

[24] Calais J., Ceci F., Eiber M., Hope T.A., Hofman M.S., Rischpler C., Bach-Gansmo T., Nanni C., Savir-Baruch B., Elashoff D. (2019). (18)F-fluciclovine PET-CT and (68)Ga-PSMA-11 PET-CT in patients with early biochemical recurrence after prostatectomy: A prospective, single-centre, single-arm, comparative imaging trial. Lancet Oncol..

[25] Meziou S., Ringuette Goulet C., Hovington H., Lefebvre V., Lavallée É., Bergeron M., Brisson H., Champagne A., Neveu B., Lacombe D. (2020). GLUT1 expression in high-risk prostate cancer: Correlation with (18)F-FDG-PET/CT and clinical outcome. Prostate Cancer Prostatic Dis..

[26] Mena E., Lindenberg M.L., Turkbey I.B., Shih J.H., Harmon S.A., Lim I., Lin F., Adler S., Eclarinal P., McKinney Y.L. (2020). (18)F-DCFPyL PET/CT Imaging in Patients with Biochemically Recurrent Prostate Cancer After Primary Local Therapy. J. Nucl. Med..

[27] Giesel F.L., Knorr K., Spohn F., Will L., Maurer T., Flechsig P., Neels O., Schiller K., Amaral H., Weber W.A. (2019). Detection Efficacy of (18)F-PSMA-1007 PET/CT in 251 Patients with Biochemical Recurrence of Prostate Cancer after Radical Prostatectomy. J. Nucl. Med..

[28] Mannas M.P., Lee T., Pourghiasian M., Wilson D.C., Black P.C. (2020). Incidentalomas of the prostate detected by 18-fluoro-2-deoxy-D-glucose positron emission tomography/computed tomography. Can. Urol. Assoc. J..

[29] Bakht M.K., Lovnicki J.M., Tubman J., Stringer K.F., Chiaramonte J., Reynolds M.R., Derecichei I., Ferraiuolo R.M., Fifield B.A., Lubanska D. (2020). Differential Expression of Glucose Transporters and Hexokinases in Prostate Cancer with a Neuroendocrine Gene Signature: A Mechanistic Perspective for ^18^F-FDG Imaging of PSMA-Suppressed Tumors. J. Nucl. Med..

[30] Sheikhbahaei S., Afshar-Oromieh A., Eiber M., Solnes L.B., Javadi M.S., Ross A.E., Pienta K.J., Allaf M.E., Haberkorn U., Pomper M.G. (2017). Pearls and pitfalls in clinical interpretation of prostate-specific membrane antigen (PSMA)-targeted PET imaging. Eur. J. Nucl. Med. Mol. Imaging.

